# Marking the 1918 influenza pandemic centennial: addressing regional influenza threats through the Asia Pacific Strategy for Emerging Diseases and Public Health Emergencies

**DOI:** 10.5365/wpsar.2018.9.5.000

**Published:** 2019-11-19

**Authors:** Erica Dueger, Lisa Peters, Li Ailan

**Affiliations:** a WHO Health Emergencies Programme, WHO Regional Office for the Western Pacific, Manila, Philippines.; b Centers for Disease Control and Prevention, Atlanta, Georgia, USA.

In 1918, near the close of the First World War, pandemic influenza swept across the world. Spread by the movement of troops and fueled by dense military-camp living quarters, the virus caused unusually high mortality rates in people 20–40 years old. An estimated 500 million people were infected, and up to 50 million died. Since then, pandemics caused by newly emerging influenza viruses have occurred every 10–40 years, with each of the pandemics in 1957, 1968 and 1977 taking the lives of roughly one million people. (*1*) More recently, the 2009 H1N1 influenza pandemic resulted in an estimated half a million deaths and raised concerns about how prepared the global community was to cope with future public health events. (*2*) Past pandemics can teach us important lessons about preventing and responding to emerging global health threats. This special issue highlights significant achievements across the Western Pacific Region in global pandemic preparedness and response.

The World Health Organization (WHO) Western Pacific Region is a hotspot for significant emerging infectious disease events, including human infections with avian influenza viruses. (*3*) Home to nearly 1.9 billion people and 6 billion poultry, avian influenza viruses that pass from animals to humans living in close proximity could mutate and rapidly spread through the Region and the world. Since 2003, the Western Pacific Region has experienced the emergence of influenza A(H1N1)pdm09, A(H5N1), A(H5N6), A(H6N1), A(H7N4), A(H7N9), A(H9N2) and A(H10N8) viruses. (*4*) Member States’ abilities to quickly identify emerging infections, determine the pandemic potential of the causative viruses, assess public health risk and event severity, and, when needed, mobilize a public health response is critical to better protecting people from emerging threats in the Region and around the world.

**Figure 1:**
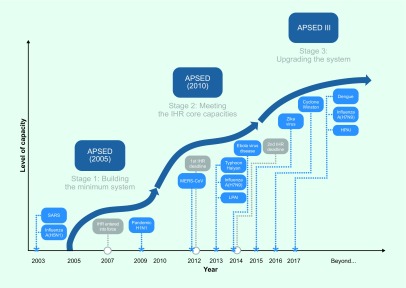
**APSED development and priorities from 2005 to 2016 and beyond**

**Figure 2:**
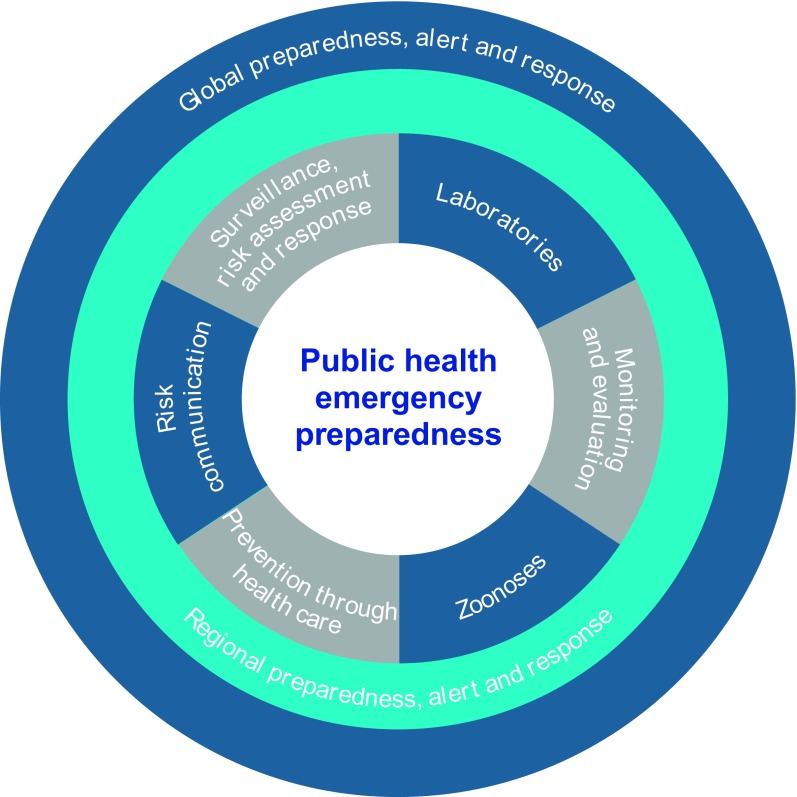
**APSED III provides a framework for public health emergency preparedness in the Western Pacific Region, including preparedness for future pandemic influenza**

For more than a decade, the *Asia Pacific Strategy for Emerging Diseases and Public Health Emergencies* (APSED III) and its earlier versions in 2005 and 2010 have driven joint efforts to build and strengthen national core capacities as required under the International Health Regulations or IHR (2005). (*5*, *6*) APSED III envisions a region able to prepare for, detect and respond to public health emergencies through improved regional connectivity and collective responsibility for managing health security. Importantly, APSED III builds on the foundations of the earlier versions to address emerging disease threats and public health emergencies (**Fig. 1**). APSED III provides critical elements for developing public health systems capable of identifying and responding to emerging infectious diseases, events and public health emergencies, including the next influenza pandemic (**Fig. 2**).

[**insert** Figure 1]

[**insert** Figure 2]

Over the last decade, Member States in the Western Pacific Region have substantially strengthened national virological and epidemiological surveillance for influenza. (*7*) Through these improved capacities, the Region contributes to the continuous global monitoring of seasonal and emerging influenza viruses through the Global Influenza Surveillance and Response System. (*8*) Event-based surveillance became a regional priority as part of the implementation of IHR (2005) and, through APSED III, is now well established in the Western Pacific Region. Outpatient influenza-like illness surveillance across the Asia-Pacific is used to evaluate seasonal severity (*9*) of influenza and provides isolates to support seasonal influenza vaccine development. (*10*) These activities are helping countries detect, conduct risk assessments of and respond to influenza outbreaks as well as contribute to biannual recommendations for vaccine composition.

The importance of coordinating pandemic preparedness and response efforts with the animal and environmental sectors cannot be overstated. Zoonotic influenza virus mutations have been associated with pandemics over the last century, and their significance was recognized broadly in 1997, when the first human cases of A(H5N1) were detected in Hong Kong Special Administrative Region SAR (China). As outlined in the perspective by Peters et al. (*11*) and overview by Hamid et al., (*4*) infected animals and contaminated environments are often the source of human infection. Close monitoring of domestic animals and wildlife, and any associated human populations, is important for identifying newly emerging influenza viruses. Sharing these data in a timely manner allows policy-makers and public health officials to quickly identify and respond to such emerging threats. In response to this need, the WHO Regional Office for the Western Pacific has developed a set of online interactive influenza dashboards (*12*) that provide both baseline seasonal and avian influenza data and real-time surveillance information for risk assessments. In addition, the WHO Regional Office has supported national pandemic containment exercises that encourage multisectoral collaboration and improve national pandemic preparedness plans. (*13*)

Risk communication is essential to moblize an effective public health response to influenza. The 2009 H1N1 influenza pandemic highlighted the importance of comprehensive risk communication strategies; the lessons learnt were applied for timely and transparent risk communication after discovery of the first human case of influenza A(H7N9) in China. (*14*) Although significant progress has been made in risk communication over the last decade, there is still room for improvement. Efforts under way in Australia highlight improvements in risk communication for in Australian Aboriginal communities (*15*) and secondary school students. (*16*)

Lessons learnt from the 2009 pandemic mobilized the World Health Assembly to adopt the Pandemic Influenza Preparedness (PIP) framework in 2011, enabling efficient and equitable access to vaccines and medicines during future pandemics. (*17*) Critical to these efforts is utilization of national surveillance data to support Member State policies and systems for seasonal influenza vaccination of high-risk groups. Determination of national disease burden, as described in detail for recent efforts in Cambodia (*18*) and China, (*19*) is imperative for obtaining national funding to support vaccination of high-risk groups and for influenza vaccination systems that can be scaled up quickly in the face of a pandemic. Collaborative efforts of PIP and the Partnership for Influenza Vaccine Introduction continue to support increased pandemic readiness through the expansion of national seasonal influenza vaccination programmes in low- and middle-income countries. (*10*)

WHO has been working with partners to ensure strong regional systems are in place to support the rapid detection, identification, reporting and risk assessment of any events with pandemic potential in the Western Pacific Region. As reflected in published influenza profiles (*20*) of the laboratory, surveillance and vaccination capacities for 37 countries and areas, influenza preparedness is well documented across the Region, including for surveillance and vaccination of high-risk groups. Under the guidance of APSED III, Member States have prioritized regional and global health security, learning from the past, engaging in the present and preparing for the future. This special issue highlights the Region’s collective journey in pandemic influenza preparedness and its significant progress over the last decade to improve health security in the Region and the world.
